# Inflammation and Oxidative Stress in Chronic Kidney Disease—Potential Therapeutic Role of Minerals, Vitamins and Plant-Derived Metabolites

**DOI:** 10.3390/ijms21010263

**Published:** 2019-12-30

**Authors:** Shara Francesca Rapa, Biagio Raffaele Di Iorio, Pietro Campiglia, August Heidland, Stefania Marzocco

**Affiliations:** 1 Department of Pharmacy, School of Pharmacy, University of Salerno, Via Giovanni Paolo II 132, I-84084 Fisciano, SA, Italy; srapa@unisa.it (S.F.R.); pcampiglia@unisa.it (P.C.); 2 UOC Nephrology AORN “San Giuseppe Moscati”, C.da Amoretta, 83100 Avellino, Italy; br.diiorio@gmail.com; 3 European Biomedical Research Institute of Salerno, Via De Renzi 50, I-84125 Salerno, Italy; 4 Department of Internal Medicine and KfH Kidney Center, University of Würzburg, KfH Kidney Center Würzburg, 97080 Würzburg, Germany; august.heidland@t-online.de

**Keywords:** chronic kidney disease (CKD), inflammation, oxidative stress, uremic toxins, minerals, vitamins, plant-derived metabolites

## Abstract

Chronic kidney disease (CKD) is a debilitating pathology with various causal factors, culminating in end stage renal disease (ESRD) requiring dialysis or kidney transplantation. The progression of CKD is closely associated with systemic inflammation and oxidative stress, which are responsible for the manifestation of numerous complications such as malnutrition, atherosclerosis, coronary artery calcification, heart failure, anemia and mineral and bone disorders, as well as enhanced cardiovascular mortality. In addition to conventional therapy with anti-inflammatory and antioxidative agents, growing evidence has indicated that certain minerals, vitamins and plant-derived metabolites exhibit beneficial effects in these disturbances. In the current work, we review the anti-inflammatory and antioxidant properties of various agents which could be of potential benefit in CKD/ESRD. However, the related studies were limited due to small sample sizes and short-term follow-up in many trials. Therefore, studies of several anti-inflammatory and antioxidant agents with long-term follow-ups are necessary.

## 1. Introduction

Renal failure is a major health issue, which has been increasing worldwide [1]. Kidney diseases include acute kidney injury and chronic kidney disease (CKD). Acute kidney injury is a reversible condition, which may progress to end-stage renal disease (ESRD), whereas CKD is a chronic condition characterized by proteinuria, a normal or reduced glomerular filtration rate (GFR) and progressive glomerular, tubular and interstitial damage. CKD is a global health issue with an increasing estimated prevalence of 8–16% [2,3]. 

The main causes of CKD are hypertension, diabetes, advanced age, immune-mediated diseases, glomerulonephritis, tubulo-interstitial disease and hereditary kidney diseases. CKD is a devastating condition which may advance to ESRD, requiring renal replacement in the form of dialysis or kidney transplantation. As dialysis allows for only partial correction of the uremic state, renal transplantation is the therapy of choice; however, it requires life-long immune suppression [4].

High levels of metabolic end-products—the uremic toxins—have become clinically relevant in CKD progression and are tightly related to many CKD-associated complications [5,6,7,8,9,10,11]. CKD patients tend to suffer from many complications, such as hypertension, cardiovascular diseases, anemia, metabolic acidosis [12], altered immune response, mineral and bone disturbances and neurological complications [13]. Among these complications, cardiovascular dysfunctions and infections promoted by an altered immune response have been shown to be responsible for an increased risk of morbidity and mortality [14]. In these conditions, inflammation and oxidative stress play pivotal roles [15].

The progression of CKD has been shown to result in inflammation and oxidative stress [16]. In fact, CKD patients typically suffer from chronic inflammation [17] and have severely impaired antioxidative systems, which worsen progressively with the degree of renal failure [18]. Inflammation and oxidative stress are crucial as defense mechanisms against infections but, if not properly regulated, they may initiate a number of deleterious effects, such as cytokine overproduction and an increase in pro-inflammatory and oxidative stress mediators [19]. Thus, the treatment of inflammation and oxidative stress is of primary importance in the uremic syndrome.

## 2. The Key Role of Inflammation in CKD

Inflammation is characterized by an increase of inflammatory markers, including cytokines, acute phase proteins and adhesion molecules, in which the cells of the innate immune response system are mainly involved. Many factors contribute to the chronic inflammatory state in CKD, including the increased production of proinflammatory cytokines and oxidative stress, as well as acidosis, chronic and recurrent infections, intestinal dysbiosis and altered adipose tissue metabolism [20]. Systemic evaluation of these markers should be performed, even in early stages of the disease. In fact, multiple lines of evidence have supported a direct pathogenic role for inflammation in CKD. Clinical studies have demonstrated that inflammatory markers are associated with many complications during CKD, such as malnutrition, coronary artery calcification, atherosclerosis, atrial fibrillation, left ventricular hypertrophy, heart failure and enhanced CKD mortality [21,22,23]. Moreover, inflammation contributes to the progression of CKD, insulin resistance, oxidative stress, endothelial dysfunction, mineral and bone disease [24,25,26], anemia [27] and erythropoietin (Epo) resistance [28]. The effects of inflammation on Epo resistance in CKD are multiple, involving decreased Epo production, a lowered effect of Epo on erythropoiesis and functional iron deficiency due to increased production of hepcidin, which impairs cellular iron release [29]. Inflammation is also a great problem in pediatric patients with CKD/ESRD [30]. 

**Figure 1:**
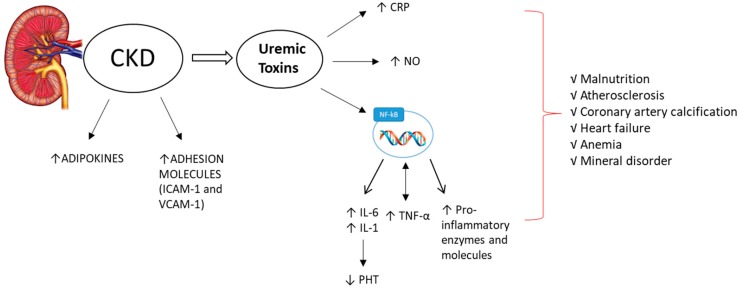
During chronic kidney disease (CKD), increases (↑) in adipokines and in adhesion proteins (such as ICAM-1 and VCAM-1) are observed. Uremic toxins play a very important role in the onset and progression of the inflammatory state, by increasing C-reactive protein (CRP), nitric oxide (NO) and a marked activation of the nuclear factor kappa-light-chain-enhancer of activated B cells (NF-κB), which lead to increased levels of pro-inflammatory cytokines, such as interleukin-6 and -1 (IL-6, IL-1). These also suppress (↓) parathyroid hormone (PTH) secretion, as well as enhancing the levels of tumor necrosis factor-α (TNF-α) and pro-inflammatory enzymes and molecules. This process leads to an exacerbation of the inflammatory state and is responsible for the onset or aggravation of various complications, such as malnutrition, atherosclerosis, coronary artery calcification, heart failure, anemia and mineral disorders.

Important markers of inflammation in CKD are C-reactive protein (CRP), interleukin-6 (IL-6), interleukin-1 (IL-1), tumor necrosis factor-α (TNF-α), adipokines, adhesion molecules and the CD40 ligand, which have been particularly implicated in the progression of CKD (Figure 1). The inflammatory marker CRP has been linked to malnutrition, atherosclerosis, erythropoietin resistance and cardiovascular morbidity and mortality [31]. In a large multi-central international database of hemodialysis (HD) patients, CRP predicted mortality with a precision comparable to that of hypoalbuminemia and exceeding ferritin and white blood cell count [32]. 

Proinflammatory cytokines, such as IL-6, IL-1 and TNF-α, have been positively associated with the severity of CKD. These are produced by adipose tissue (in addition to being produced by lymphocytes), which becomes dysfunctional during CKD. In fact, in this condition, the visceral adipose tissue expresses a high level of mRNA of pro-inflammatory cytokines (e.g., TNF-α, adiponectin receptor-1, CD68 and monocyte chemoattractant protein-1; MCP-1) [33]. Furthermore, IL-6 contributes to the development of atherosclerosis through metabolic, endothelial and procoagulant mechanisms. Therefore, IL-6 measurement may have clinical utility as a predictive marker for atherosclerosis [34]. Pro-inflammatory markers, such as IL-6, may also be associated with depression in patients with advanced CKD. This aspect may be a predictor of morbidity and mortality in CKD patients, as it has been associated with a reduction in nutrient intake [35]. In addition, IL-1 and IL-6 have been shown to suppress secretion of parathyroid hormone (PTH). Low PTH levels in HD patients have been associated with malnutrition, inflammation and cachexia syndrome, as well as low bone turnover disease [36]. Low PTH levels may also result in increased mortality in HD patients [37]. In addition to traditional risk factors, TNF-α has also been significantly associated with an increased risk of heart failure in CKD and high levels of TNF-α have been associated with markers of malnutrition and inflammation, as well as predicting mortality [38]. Moreover, it has been shown to play a possible role in the development of diabetic nephropathy [39]. TNF-α is also one of the main activators of the receptor of the nuclear factor kappa-light-chain-enhancer of activated B cells (NF-κB) ligand (RANKL), which has been considered to be a key trigger of osteoclast activation and bone resorption and thus may also have a relationship with fractures observed in HD patients [40]. The levels of these cytokines, as well as the increase of pro-inflammatory enzymes such as cyclooxygenase-2 (COX-2) and inducible nitric oxide synthase (iNOS), are positively regulated by the activation of NF-κB in patients with CKD [41].

Numerous studies have shown that toxins released from intestinal tract, such as indoxyl sulfate (IS), p-cresol (PC) and p-cresol sulfate (PCS), are involved in the inflammatory state in CKD [42]. Uremic toxins have also been shown to contribute to many uremia-associated dysfunctions, including an altered immune response [43]. In fact, several studies have shown that uremic toxins, such as IS, increase the levels of TNF-α and IL-6 and cause an exacerbation of the inflammatory state through the promotion of oxidative stress [44]. Many studies have reported the effects of uremic toxins from the intestine. These gut-derived uremic toxins have been shown to play a pivotal role in affecting intestinal homeostasis, as well as inducing inflammation and oxidative stress in the systemic circulation [45,46]. Uremic toxins have been positively associated with NF-κB expression and exhibit positive correlations with CRP levels, iNOS and COX-2 expression [43,47]. It has been shown that IS induces marked neuroinflammation, which is responsible for the neurodegenerative disorders associated with CKD [48]. Neuroinflammation is characterized by a marked increase in pro-inflammatory cytokines, such as TNF-α, caused by an increase in the expression of enzymes involved in the mechanisms of inflammation (e.g., COX-2 and iNOS), nitric oxide (NO) levels and the nuclear translocation of NF-κB [48]. Uremic toxins have been strongly implicated in the manifestation and progression of the inflammatory state associated with CKD, by modulating a series of mediators such as CRP, cytokines and transcription factors.

Recently, the role of adipochines in CKD has received a lot of attention in scientific research. In particular, studies of the pro-inflammatory agents leptin, apelin, omentin, visfatin and resistin, as well as the anti-inflammatory adiponectin, have demonstrated that CKD is associated with higher leptin and adiponectin levels, as determined from the National Health and Nutrition Examination Survey (NHANES) [49,50]; showing that means of reduction of such adipokines, such as weight reduction in obese patients, may improve GFR in those at risk of CKD [51].

For these reasons, these biomolecules could be useful both for risk stratification and as potential therapeutic targets. 

Adhesion molecules (ICAM-1 and VCAM-1) are cell-surface glycoproteins induced at endothelial sites of inflammation which are responsible, in part, for the adherence of hematopoietic cells to the endothelium. Adhesion molecules are upregulated in CKD patients as a consequence of both decreased clearance and enhanced synthesis. This upregulation is currently considered to be an expression of endothelial dysfunction in CKD patients [52].

## 3. Oxidative Stress and CKD

**Figure 2:**
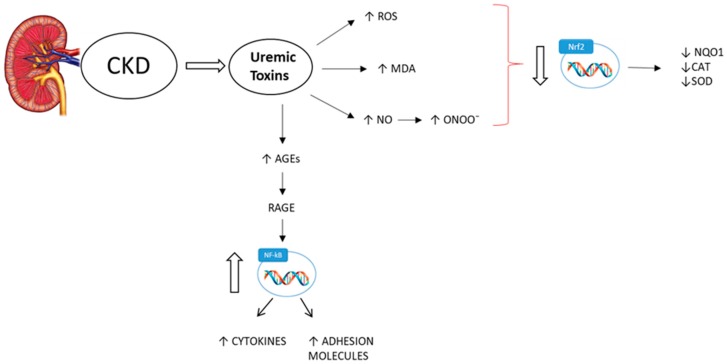
During chronic kidney disease (CKD), there is a considerable increase in oxidative stress due to uremic toxins. There are increases in reactive oxygen species (ROS), nitric oxide (NO) and markers of oxidative stress—such as malondialdehyde (MDA), peroxynitrite (ONOO^−^) and advanced glycation end products (AGEs), which interact with AGE receptors and determine the activation of the nuclear factor kappa-light-chain-enhancer of activated B cells (NF-κB), with a consequent increase in cytokines and adhesion molecules. The oxidative stress condition is reflected in a reduction in the activity of the transcription factor nuclear factor erythroid 2-related factor 2 (Nrf2) and, consequently, of the expression of antioxidant and cytoprotective enzymes such as NAD(P)H dehydrogenase [quinone] 1 (NQO1), catalase (CAT) and superoxide dismutase (SOD).

Oxidative stress is frequently observed in CKD/ESRD and is a non-traditional risk factor for all causes of mortality [53,54]. For this reason, oxidative stress has become an important diagnostic and prognostic factor and is a target for CKD prevention/treatment. High levels of oxidative stress have already been found in the early stages of CKD [55], which increase in parallel with the progression to ESRD [56]; and is further exacerbated in HD patients [57,58]. ESRD patients on peritoneal dialysis (PD) have increased oxidative stress, when compared to non-dialysized uremic patients (but lower, when compared to HD patients) [57,59]. In fact, HD and PD have both been shown to increase oxidative processes, leading to an increase in the state of oxidative stress. Moreover, oxidative stress may also persist after renal transplantation. Oxidative stress has also been linked to the production of highly reactive intermediates during inflammation; on the other hand, reactive oxygen species (ROS) are able to further enhance the inflammatory response by triggering pro-inflammatory mediators (e.g., NF-κB). Low amounts of pro-oxidative agents, which have important defensive roles, are normally produced by cells but are inactivated by enzyme systems (e.g., glutathione) and other antioxidants (called scavengers) for their ability to neutralize free radicals. In the kidneys, ROS are mainly produced by the mitochondrial respiratory chain and by enzymes such as NADPH oxidase (NOX). The different NOX isoforms, including NOX1, NOX2 and NOX4, are mainly responsible for oxidative stress, worsening vascular function and promoting fibrosis. [60,61]. Recently, NOX5 expression has been observed to be increased in human biopsy samples from patients with diabetic nephropathy [62]. Excessive ROS production cannot be neutralized by scavenger systems and can cause oxidative damage to proteins, nucleic acids and lipids, as well as affecting cellular activity and inhibiting enzymatic activity. When an imbalance is established between oxidizing agents and scavenger defense systems, a condition of oxidative stress is created. The consequences of this are very dangerous; especially for nucleic acids, where modification of bases, covalent crosslinks and single- and double-strand breaks can occur. Among the bases of DNA, guanine is particularly sensitive to oxidative reactions due to radical species, leading to oxidized products including 8-hydroxy-2′-deoxyguanosine (8-OH-dG), one of the most abundant oxidative products of nucleic acids [63]. This damage appears to be involved in a variety of chronic and degenerative diseases, such as CKD. In CKD patients, elevated levels of oxidative stress have been observed, due to the impairment of their physiological defense mechanisms. This can cause oxidative damage to nucleic acids, resulting in increased risk for the onset of subsequent tumors [64]. In addition to the radical species deriving from oxygen, other radicals are derived from reactive nitrogen species (RNS). Among these, the superoxide anion (O_2_^−^) is the major free radical generated in vivo by the reduction of molecular oxygen through the action of the NOX enzyme complex. As soon as O_2_^−^ is formed, it is converted into hydrogen peroxide (H_2_O_2_). Excessive production of ROS by NADPH oxidase is commonly thought to be responsible for the tissue injury associated with a range of chronic inflammatory diseases and has long been considered a unique property of phagocytic cells. Both O_2_^−^ and H_2_O_2_ are precursors for the production of more powerful oxidants. O_2_^−^ has a high affinity for reacting with the free radical NO, which rapidly produces the RNS peroxynitrite (ONOO^−^). ONOO^−^ and hydroxyl (OH^−^) can lead to extensive nitrosative and oxidative modifications to proteins, lipids and nucleic acids. Several markers of oxidative stress, such as malondialdehyde (MDA), oxidized low-density lipoprotein, advanced glycation end products (AGEs) and 8-hydroxyde-oxyguanosine, have significantly elevated levels in circulating blood and/or tissue in CKD patients [65]. AGEs interact with cells through a specific receptor system for AGEs (RAGE) [66]. The interaction between AGEs and the RAGE receptor induces the activation of the MAP kinase transduction pathway, determining nuclear translocation of the p65 subunit of NF-κB and the activation of second messengers, with a consequent increase of cytokines, pro-inflammatory enzymes and adhesion molecules [67]. This is largely caused by the impaired activation of nuclear factor erythroid 2-related factor 2 (Nrf2), the transcription factor which regulates genes encoding antioxidant and detoxifying proteins and enzymes, such as superoxide dismutase (SOD), catalase (CAT) and NAD(P)H dehydrogenase [quinone] 1 (NQO1) [68]. At the renal level, oxidative stress is responsible for progressive renal damage, which can lead to renal ischemia, lesions to the glomeruli, cell death and apoptosis, exacerbating the severe inflammatory processes already underway. Among other things, oxidative stress is also responsible for several pathological conditions considered as risk factors for CKD, such as diabetes, hypertension and atherosclerosis [69]. In fact, reduction of the bioavailability of NO caused by endothelial dysfunction induced by oxidative stress favors the progression of atherosclerosis. Specifically, the accumulation of ROS (especially O_2_^−^) leads to the inactivation and deficiency of NO, which is a critical antioxidant protecting kidney function by increasing renal blood flow, enhancing pressure natriuresis, regulating tubuloglomerular function and preserving fluid and electrolyte homeostasis. NO deficiency and high levels of plasma O_2_^−^ are considered critical promoters of oxidative stress. Antioxidant therapies may be beneficial in reducing oxidative stress, lowering uremic cardiovascular toxicity and improving survival [70] (Figure 2). Even at the onset of oxidative stress, uremic toxins have a predominant role. In fact, recent evidence has shown that IS-increased ROS production is generated mainly through activation of nicotinamide adenine dinucleotide phosphate (NADPH) oxidase [44]. Moreover, it has been reported that IS enhances inflammatory response and ROS in LPS-stimulated macrophages [43]. In addition, it has been reported that uremic toxins are responsible for the neurodegenerative disorders characteristic of CKD through an increase in oxidative stress in the glia cells, increases of ROS and oxidant markers (such as MDA [71] and ONOO^−^) and downregulation of the transcription factor Nrf2 as well as expression of enzymes related to the cytoprotective and antioxidant activities associated with it [48].

## 4. Anti-Inflammatory and Antioxidant Compounds

In CKD patients, lifestyle factors, such as aerobic exercise and dietary interventions [72,73], have been shown to exert anti-inflammatory effects; however, the adherence for CKD patients is often poor, thus leading to pharmacological therapy as a potential alternative. The use of statins [74] and angiotensin-converting enzyme inhibitors, as well as AT1-blockers, have been shown to exert some anti-inflammatory effects [75], even if some studies have suggested their inefficiency in dialysis patients [76,77,78,79]. However, in addition to the conventional therapy, the use of supplements has gathered interest in scientific research. Despite the involvement of oxidative stress, antioxidant therapies have not become a standard of care in CKD patients to date and more investigations are needed. Numerous studies have shown the possibility of using compounds with anti-inflammatory and antioxidant activities in the treatment of CKD. In particular, several classes of vitamins and minerals, as well as plant-derived metabolites, are of growing interest.

### 4.1. Minerals and Vitamins

Patients with CKD show weakened antioxidative defense mechanisms, in part due to dietary restrictions on fruits and vegetables resulting in lower levels of vitamins C and E. In addition, vitamin C is lost during dialysis treatment, as well as selenium levels; furthermore, the function of the glutathione (GSH)-scavenging mechanism is reduced. Endogenous or dietary antioxidants are protective against oxidation, inflammation and kidney damage in CKD (Table 1). 

#### 4.1.1. Magnesium 

Magnesium is one of the most important cations in the human body. The kidney plays a major role in regulating the magnesium balance and homeostasis—70% of the circulating magnesium is filtered by the glomerulus and approximately 90–95% of the filtered magnesium is reabsorbed [80]. When the renal function declines, the ability for renal excretion deteriorates [81]. Low levels of magnesium are associated with several complications, such as hypertension and vascular calcification and are also associated with an increased risk for both cardiovascular disease and non-cardiovascular mortality [82]. Given that cardiovascular disease and CKD share similar risk factors, a low magnesium status may also contribute to the progression of CKD. In cohort studies in HD, PD and CKD patients, lower serum magnesium levels have been associated with an increased risk of all-cause and/or cardiovascular mortality [83]. The ability of magnesium to reduce the risk of cardiovascular complications during CKD is also due to its anti-inflammatory properties, which involve reducing CRP levels [84]. Magnesium deficiency should be supplemented by the administration of magnesium salts. In moderate CKD, increases in the fractional excretion of magnesium largely compensate for the loss of glomerular filtration rate in maintaining normal serum magnesium levels. However, in advanced CKD (stage 4–5), these compensatory mechanisms become inadequate and the fraction of filtered magnesium excreted increases as a result of impaired tubular reabsorption. This becomes even more marked when the GFR falls below 10 mL/min. Thus, the compensatory rise in magnesium excretion is insufficient to prevent an increase in serum magnesium concentration. As a consequence, ESRD is the only clinical condition in which sustained hypermagnesemia may occur and net magnesium balance may be positive [85]; which is probably related to normal gastrointestinal absorption and impaired net kidney excretion. Generally, magnesium should be measured regularly, and dialysate magnesium adjusted accordingly, in order to maintain plasma magnesium within the normal range.

#### 4.1.2. Selenium

Selenium is an essential micronutrient and its consumption depends on its soil levels and the resulting food content. In many populations in Western Europe, selenium blood levels are lower than those in North America. Severe selenium deficiency leads to the development of Kashin–Beck disease, characterized by muscle loss and cardiomyopathy [86]. In CKD and ESRD, selenium deficiency is common due to decreased intestinal absorption and/or loss during dialysis treatment. Selenium is a cofactor for glutathione peroxidases (GSH-Px), which are synthesized in the kidney and play an important role in ROS metabolism. In CKD and ESRD, the formation of GSH-Px is reduced but can be treated with Selenium supplementation. Kidney transplantation rapidly restores plasma GSH-Px [87].

#### 4.1.3. Phosphate

Phosphate homeostasis is regulated by an integrated mechanism involving the kidneys, bones, intestines and muscles [88]. In CKD, multiple aspects of phosphate homeostasis are altered; each of which being associated with higher mortality risk, coronary artery calcification, vascular stiffness, progression of CKD and ESRD [89]. Serum concentrations of the phosphaturic hormones, PTH and fibroblast growth factor 23 (FGF-23) are markedly increased in CKD; likely in response to dysregulated phosphate handling [90]. Considering the consistent associations between hyperphosphatemia with morbidity and mortality in CKD, there is a need for lowering phosphate in clinical practice. Interestingly, although phosphate binders have been widely used for phosphate lowering in CKD patients and are recommended for use in CKD by the Kidney Disease Improving Global Outcomes (KDIGO) guidelines, they have been approved by the Food and Drug Administration (FDA) only for use in ESRD patients [91]. Moreover, nutritional therapy without animal proteins (e.g., when replaced by vegetal proteins, such in as the Very Low Protein Diet; VLPD) can reduce phosphorus levels as well as FGF23 and PTH [46,92,93,94].

#### 4.1.4. Vitamin C

Ascorbic acid has attenuated oxidative damage, inflammation and renal injury in several animal models and in nephrotoxic acute kidney injury, ischemia and rhabdomyolysis-induced renal injury [95]. In fact, deficiency of vitamin C—an abundant non-enzymatic antioxidant—is prevalent in hemodialysis as a result of dietary restrictions and/or loss during dialysis [96]. Vitamin C cannot be synthesized endogenously but is taken through diet. For this reason, there is a reduction in plasma levels of this vitamin during CKD, due to the dietary restrictions on the intake of fruits and vegetables imposed in order to avoid hyperkalaemia. Vitamin C deficiency may be associated with adverse cardiovascular outcomes [97]. Deicher et al. observed that a low plasma vitamin C level predicted fatal and major non-fatal adverse cardiovascular events among HD patients [96]. Renal dysfunction has been associated with a decreased level of plasma vitamin C in patients with CKD [98]. Increased oxidative stress due to vitamin C deficiency can also lead to endothelial dysfunction, through low-density lipoprotein oxidation, in non-diabetic patients with CKD [98]. Vitamin C has an important antioxidative function, being able to reduce ROS levels. This provides protection against kidney oxidative damage, as well as maintaining vascular and endothelial function due to the ability of vitamin C to maintain hydroxylase and mono-oxygenase enzymes [99]. Supplementation of ascorbic acid may be particularly important in acute renal failure patients with low vitamin C status. Moreover, vitamin C is also easily oxidized to dehydro-ascorbic acid during hemodialysis. Indications for ascorbate supplementation have been formed very cautiously and, at present, the recommended doses might not be optimal and may not meet real requirements [78]. However, the application of large vitamin C doses is risky, as the metabolism of ascorbic acid leads to a large concentration of oxalic acid in plasma and in soft tissues; moreover, oxalate levels in dialysis patients are elevated. Therefore, CKD and ESRD patients should have a limited daily vitamin C supplement of 75 mg for females and 90 mg for males [100]. 

#### 4.1.5. Vitamin E

Vitamin E is a fat-soluble vitamin. Its main active compound is α-tocopherol. Vitamin E is a potent antioxidant with anti-inflammatory properties, which particularly interferes with cell membrane lipid peroxidation [101]. In experimental studies, including an investigation in cats, vitamin E has been shown to be a potential antioxidant and to slow atherosclerosis [102]. Furthermore, in observational clinical studies, vitamin E consumption of more than 100 IU a day lowered the rate of coronary events. However, in the HOPE trial, in patients with high risk of cardiovascular events, vitamin E supplementation in daily doses of 400 IU had no apparent effect on cardiovascular outcomes [103]. In addition, in patients with mild to moderate renal insufficiency and a high cardiovascular risk, it had no apparent effect on cardiovascular outcomes [104]. The subsequent extension of this study (the HOPE TOO trial), unfortunately, was followed by enhanced cardiovascular complications in the vitamin E-treated patients [105]. 

In ESRD, vitamin E levels have been found to be low, normal or increased [100]. The lower levels were obviously caused by decreased dietary intake. In a randomized prospective investigation (SPACE study) in hemodialysis patients, supplementation of α-tocopherol (800 IU) led to a significant improvement of cardiovascular complications [106]. In another study with a small number of dialysis patients, the beneficial effects of α-tocopherol therapy have been described [107]. Besides oral application, vitamin E-coated membranes have been used to lower oxidative stress during hemodialysis and various beneficial effects were reported [108]. 

#### 4.1.6. Vitamin D

Vitamin D is not only important for the homeostasis of calcium/phosphorus and skeletal health but also for numerous extra-skeletal functions. Vitamin D deficiency (serum 25(OH)D levels < 20 ng/mL) and insufficiency (serum 25(OH)D levels < 30 ng/mL) have been observed in numerous diseases in the general population [109]. In particular, vitamin D deficiency has been frequently observed in CKD and ESRD. The serum levels appear to have an inverse relationship with kidney function and a particular prevalence in hemodialysis patients [110]. Growing evidence has indicated that vitamin D deficiency may contribute to deteriorating renal function, as well as increased morbidity and mortality in patients with CKD [111]. In a rat model, vitamin D deficiency has been shown to enhance contrast-induced nephropathy, a frequent cause of the acute renal failure in hospitals [112]. In diabetic nephropathy, application of vitamin D ameliorated the kidney podocyte function and decreased proteinuria [113]. In other pre-clinical studies, vitamin D has attenuated kidney injury by suppressing fibrosis, inflammation and apoptosis, through the inhibition of multiple key pathways in kidney injury, such as the renin-angiotensin-aldosterone system (RAAS), NF-κB, TGF-β/Smad and Wnt/β-catenin signaling pathways [114,115,116,117].

#### 4.1.7. Vitamin A

Vitamin A homeostasis is altered in adults with CKD. It has been shown that all-trans retinoic acid (ATRA) has beneficial effects on early inflammation in glomeruli and proximal and distal tubules in streptozotocin-induced diabetes; ATRA decreased inflammatory response. In fact, ATRA administration attenuated TLR4/NF-κB inflammatory signaling and prevented NF-κB nuclear translocation in glomeruli and proximal tubules [118].

#### 4.1.8. Vitamin B1 (Thiamin)

Vitamin B1 is an indispensable nutrient, where its deficiency has been associated with central and cardiovascular disturbances. In plasma, it is mainly bound to albumin. Thiamine-pyrophosphate is a coenzyme particularly involved in the metabolism of carbohydrates. In ESRD, its plasma concentration has been found to be either normal, decreased or elevated [119,120]. Functional thiamine deficiencies are frequently observed, even at elevated plasma levels. According to newer investigations, such functional thiamine deficiency can be explained by a high plasma concentrations of the thiamine antimetabolite (oxythiamine) [121]. With regards to such functional thiamine deficiencies, patients on HD and PD should get a daily dose of 1.1–1.2 mg/day.

Disturbances of thiamine are very common in diabetic patients. Its treatment is performed frequently with Befotiamine, which is a lipid-soluble derivative of thiamine. Its lipid solubility is much higher and, thus, it can penetrate nerves more readily. It has been shown to improve the macro- and micro-vascular endothelial dysfunction caused by individual type 2 diabetes. In a study of hemodialysis patients, efotiamine improved the antioxidative capacity of the plasma and ameliorated the frequent genomic damage in peripheral lymphocytes [122] (Table 1).

### 4.2. Plant-Derived Metabolites

In addition to endogenous antioxidants, several dietary plant metabolites, including quercetin, curcumin, resveratrol and others, appear to be efficacious in CKD. Several natural-derived agents have been used in Chinese herbal medicine, including flavonoids (or bioflavonoids), which form an important group of secondary plant metabolites (Table 2). Many studies have provided evidence supporting the importance of nutrition during CKD; in particular, the role of nutrition in regulating the levels of gut-derived uremic toxins has been demonstrated to be beneficial in CKD patients [45,123,124,125,126].

Quercetin is an important flavonoid, which has been reported to exhibit several biological activities which include (but are not limited to) antioxidant, antidiabetic and anti-inflammatory activities [127,128]. Quercetin reduced the levels of FGF23, PTH, inorganic phosphate and urinary protein-to-creatinine ratios and urinary uric acid, creatinine and blood urea nitrogen, as well as increasing the expression of serum lactate dehydrogenase (LDH), SOD and total antioxidant activity, in an adenine-induced CKD rat model [129]. Moreover, quercetin treatment in CKD rats reduced abnormal histopathological renal changes, including chronic interstitial inflammation [130].

Curcumin is a bright yellow–orange vegetable pigment. It is abundantly represented in the tuberized rhyzo (root) of various species of *Curcuma*, especially in that of *Curcuma longa*. The roots of *Curcuma* have been widely used in Indian and Asian cuisine to prepare curries and various typical local sauces. In Ayurvedic medicine, thanks to their curcumin content, they have been used, for many centuries, in the treatment of a wide variety of disorders. In the food and cosmetics industry, curcumin is a yellow–orange coloring food additive. The most interesting properties of curcumin—as they are potentially useful in the treatment of a fairly wide range of pathologies—are their antioxidant and anti-inflammatory properties. The antioxidative properties of curcumin are attributable to their ability to react directly with radical species and to increase the gene expression of proteins with cytoprotective and antioxidant activities [131]. The protective effects of curcumin on kidney damage have been related to the downregulation of profibrotic cytokines, vascular endothelial growth factor (VEGF), TGF-β, connective tissue growth factor (CTGF) and osteopontin, as well as in extracellular matrix proteins, such as fibronectin and collagen IV [132]. In addition, curcumin has been shown to be able to reduce the progression of structural kidney damage by reducing the glomerulosclerosis index, tubulointerstitial fibrosis and arteriolopathy [130]. Synthetic analogues of curcumin, such as C66, have been shown to improve diabetic nephropathy [131]. Curcumin treatment has been shown to reduce the inflammatory kidney response by the attenuation of renal macrophage infiltration and expression of iNOS, COX-2 and proinflammatory cytokines such as TNF-α and MCP-1. The anti-inflammatory effects of curcumin have been associated with the inhibition of c-Jun N-terminal kinase (JNK)/NF-κB activation [133]. The antioxidant properties of curcumin can be traced back to its scavenger properties. In fact, it is able to neutralize radical species, such as the superoxide anion and NO. These activities are likely due to the presence of phenolic groups in the chemical structure of curcumin [134]. In addition to its direct scavenging properties, curcumin also has an indirect antioxidative activity, due to its ability to determine the upregulation of enzymes and cytoprotective and antioxidant proteins, such as SOD, CAT, NQO1 [135], glutathione S-transferase [136] and γ-glutamylcysteine ligase [137]. Moreover, the antioxidant and renoprotective properties of curcumin have been identified in 5/6 nephrectomized rats. Under these conditions, curcumin reversed oxidative stress and glomerular hemodynamic alterations; induced Nrf2 nuclear translocation; prevented glomerular hypertension, hyperfiltration and oxidative stress; and improved the expression of antioxidant enzymes [138,139]. In the same experimental model, curcumin prevented the disturbance of mitochondrial dynamics. In fact, the preservation of mitochondrial functions, bioenergetics and prevention of oxidative stress improve renal function [140]. Moreover, curcumin has been shown to ameliorate CKD-induced cardiac fibrosis and diastolic dysfunction by suppressing NLRP3 inflammasome activation [141]. Another important anti-inflammatory activity attributed to curcumin is improvement of the barrier function of the intestinal epithelium, positively modulating the expression of intestinal alkaline phosphatase and tight junction proteins and correcting gut permeability [142]. In fact, there is generally an increase in intestinal permeability during CKD, which results in the passage of pro-inflammatory molecules from blood to gut. This action reduces the levels of circulatory inflammatory biomolecules, which may be useful in the treatment of CKD. 

Resveratrol (3,5,4′-trihydroxy-trans-stilbene) is a stilbenoid (a type of natural phenol) and a phytoalexin, which is produced by several plants in response to injury or when under attack by pathogens (e.g., bacteria or fungi). Sources of resveratrol in food include the grape skin, blueberries, raspberries, mulberries and peanuts. It has anti-inflammatory, antioxidant and vaso-protective properties. Its antioxidant activity is due to the inhibition of lipid peroxidation, the ability of chelate metal ions and the direct action of radical scavenging. Moreover, resveratrol enhances antioxidant enzyme production and modulates nuclear factors involved in inflammation and oxidative stress, such as Nrf2 and NF-κB [143]. In fact, NF-κB activation plays a key role in the development and progression of CKD and its related disorders [144]. In contrast to NF-κB, Nrf2 is responsible for the expression of antioxidant and cytoprotective enzymes and is the main defense against cellular damage related to oxidative stress [145]. Due to its anti-inflammatory and antioxidant effects, resveratrol has been widely tested in animal models of chronic renal diseases and related disorders. In mice, Liang et al. suggested that resveratrol treatment inhibits oxidative stress and renal interstitial fibrosis [146]. Recently, in a mouse model of nephropathy associated with obesity induced by a high-fat diet, Cheng et al. showed that resveratrol treatment could alleviate renal damage by suppressing inflammation and oxidative stress [147]. In streptozotocin-induced diabetic rats, resveratrol also protected the kidneys by attenuating hyperglycemia-mediated oxidative stress and renal inflammatory cytokines by Nrf2–Keap1 signaling [148]. Moreover, resveratrol treatment attenuated CKD-induced skeletal muscle atrophy through the MuRF1 signaling pathway [149]. These satisfactory results may suggest the clinical use of resveratrol as a supplement to the treatment of CKD. However, no studies have yet been conducted on CKD patients in order to better clarify its side effects, probably due to its low availability. 

Cordycepin (3′-deoxyadenosine) is a derivative of the nucleoside adenosine, differing from the latter by the absence of the hydroxy group in the 3′ position of its ribose moiety. It was initially extracted from the fungus *Cordyceps militaris* but has also been produced synthetically. Cordycepin has been widely used in traditional Chinese and Tibetan medicine for its anti-inflammatory effects and health benefits [150], including its anti-asthma effects in particular [151]. Moreover, cordycepin has been shown to improve cellular oxygen absorption, cardiovascular health and heart functionality, sexual functionality, protect the liver and has been used to treat childhood palpitations, epilepsy and convulsions [152]. The renoprotective action of cordycepin has also been observed [153,154]. In a recent clinical study, cordycepin has been shown to improve CKD by affecting the TLR4/NF-κB signaling pathway. In the study, pro-inflammatory parameters such as TLR4 levels, NF-κB activation, COX-2 expression and TNF-α and IL-1β levels were modulated by cordycepin treatment. This treatment also reduced the levels of urinal protein, blood urea nitrogen and creatinine and improved the lipid profile and oxidative stress in CKD patients [155]. In addition to cordycepin, another important component of the species of the genus *Cordiceps* is N6-(2-hydroxyethyl)-adenosine (HEA). Several studies have shown that HEA has a beneficial effect on unilateral ureteral obstruction (UUO)-induced tubulointerstitial fibrosis by suppression of inflammation and renal fibroblast activation by modulation of the NF-κB and TGF-β1/Smad signaling pathways. HEA has been shown to improve kidney injury in this experimental model, as well as reducing fibrosis-related proteins and pro-inflammatory cytokines (e.g., TNF-α, IL-6 and IL-1β) and inhibiting the TGF-β1/Smad and NF-κB signaling pathways both in vivo and in vitro [154].

Flavonoids of Coreopsis tinctoria. *C. tinctoria* is an ornamental plant widely cultivated in China and in many other counties, such as Canada, Mexico and the United States. It has been reported that *C. tinctoria* possesses many biological activities, including anti-inflammatory, antioxidant, anti-hyperlipidemic and anti-hypoglycemic activities [156,157]. Its anti-inflammatory activity has been mainly attributed to inhibition of COX-2 expression [158]. However, its antioxidant property is due to the direct effect of scavenging against radical species. Several studies have shown that *C. tinctoria*, which is rich in flavonoids, reduced damage to renal tissues in high glucose/fat diet- and streptozotocin-induced diabetic rats. *C. tinctoria* may also decrease lipid levels through the adipose differentiation-related protein (ADRP) [157]. Lipid accumulation may also induce ROS activation [159]. In addition, *C. tinctoria* reduced renal inflammation and fibrosis by inhibiting the AMPK and TGF-β/Smad signaling pathways [160]. Renal inflammation was also reduced by inhibition of the NF-κB pathway. In fact, suppression of NF-κB mediated by *C. tinctoria* reduced MCP-1 and collagen IV expression and improved renal inflammation and fibrosis [161]. 

Flavonoids and polyphenols of Phyllanthus niruri. *P. niruri* is a wide-spread tropical plant, belonging to the Euphorbiaceae family, which has been widely used in Ayurvedic medicine for the treatment of bronchitis, anemia, leprosy and asthma [162]. Many studies have shown its antioxidant activity both in vitro and in vivo [163]. In fact, its high flavonoid and polyphenol content leads to ROS scavenging and chelating transition metal ion activities, which play pivotal roles in preventing the oxidation of low-density lipoproteins and in ameliorating the inflammatory condition. Its renoprotective activity is probably attributable to ability of *P. niruri* to neutralize radical oxygen species, inhibiting the formation of peroxides and blocking the chain of oxidative reactions and protecting the kidney from oxidative damage during CKD. The abilities of *P. niruri* to improve SOD activity at the kidney level to and preserve kidney function are also very interesting. The levels of CAT and GPx, which play key roles in the mechanisms of defense against oxidative stress, have been increased by a leaf extract of *P. niruri* [164]. These activities have been supported by studies that verified the ability of other *Phyllanthus* species to protect kidney function from acetaminophen-induced damage in rats.

Allicin (allyl 2-propenethiosulfinate or diallyl thiosulfinate) is an organosulfur compound obtained from garlic, a species in the family Alliaceae. The enzyme alliinase is responsible for the conversion of the stable precursor S-allyl cysteine-S-oxide (alliin) into allicin when garlic cloves are crushed or macerated [165]. Its biological properties can be attributed to both its antioxidant activity and its reaction with proteins containing a thiol group. In some studies, allicin has demonstrated a protective activity against coronary endothelial dysfunction and right heart hypertrophy in pulmonary hypertensive rats [166]. This heart-protective effect has also been shown in a rat model of CKD. Protective activity at the kidney level, in addition to the reduction of CKD-mediated systemic hypertension, appears to be due to the antioxidative effect of allicin [167]. In fact, allicin has been shown to improve renal function by modulation of AT1, Nrf2/Keap1 and eNOS pathways. The beneficial effects demonstrated by allicin are similar or even better, than those of losartan, a drug commonly used as a first-line therapy [168]. By increasing the nuclear translocation of the Nrf2 factor, allicin also increases the gene transcription of Nrf2-related enzymes, such as CAT, SOD and GPx, which play a key role in the cellular mechanisms defending against oxidative damage. During CKD, allicin has been shown to improve the biochemical markers of renal dysfunction, such as creatinine, blood urea nitrogen, diuresis and proteinuria [167].

Ursolic acid is a pentacyclic triterpenoid which was identified in the epicuticular waxes of apples as early as 1920 and is widely found in the peels of fruits, as well as in herbs and spices (e.g., rosemary and thyme). A number of potential biochemical effects of ursolic acid have been investigated but no clinical study demonstrating its benefits for human health has been carried out. The positive results obtained in animal models with ursolic acid treatment have mainly related to the improvement of glycide and lipid metabolism, thus providing evidence that it may be useful in the treatment of diabetes and obesity [169]. Some studies have shown that ursolic acid has protective effects against CKD and renal fibrosis. This protective effect appears to be due to its anti-inflammatory properties. In particular, ursolic acid has been shown to inhibit the activation of STAT3 and the NF-κB pathway, reducing the inflammatory response in the CKD-induced decline of muscle mass with progressive protein loss [170]. Interestingly, ursolic acid has been shown to cause direct inhibition of the expression of pro-inflammatory cytokines in muscles of mice with CKD [171]. This effect has been directly related to myostatin inhibition at the muscle level [172]. Inhibition of NF-κB by ursolic acid inhibits the expression of CKD-stimulated IL-6 and, probably, other cytokines; as a consequence, ursolic acid inhibits p-STAT3 activation and C/EBP-δ transcription [173]. Ursolic acid may increase bile excretion and decrease bilirubin level [174]; high total bilirubin level may be correlated with mortality among CKD patients undergoing long-term hemodialysis [175].

Epigallocatechin-3-gallate. (-)-Epigallocatechin-3-gallate (EGCG) is one of the many polyphenols present in the green tea plant. It is also the most active and has many biological properties. In particular, several studies have highlighted the antioxidative potential of EGCG. This property is directly due to its ability to neutralize free radicals, act as ROS scavenger and to chelate metal ions [176]. Bao et al. have demonstrated its beneficial role in the treatment of kidney injury in an animal model of glomerulonephritis [177]. In another study, EGCG has been shown to reduce lupus nephritis progression in mice by enhancing the Nrf2 antioxidant pathway and inhibiting NLRP3 inflammasome activation. In particular, EGCG is able to enhance the nuclear translocation of Nrf2 and to increase GSH-Px expression. This activity is combined with its ability to inhibit p65 NF-κB translocation and NLRP3 inflammasome activation through inhibition of caspase-1, IL-1β and IL-18 [167]. EGCG has also improved the biochemical markers of renal dysfunction, such as proteinuria and serum creatinine. Many studies have shown that EGCG-treated mice showed reductions in p-Akt, p-JNK, p-ERK1/2 and p-P38, as well as an increase in PPAR-γ and sirtuin-1 levels [178,179]. In a mouse model of diabetic nephropathy, EGCG ameliorated renal injury by reducing oxidative stress markers [180]. Interestingly, EGCG also reduces levels of uremic toxins, such as methylguanidine, by inhibition of oxidative stress in renal damage induced in rats [181]. Ultimately, EGCG has proven to be able to protect the kidneys from ischemia reperfusion injuries by heme oxygenase 1 upregulation and to inhibit macrophage infiltration, both of which are responsible for the progression of kidney damage [182].

## 5. The Importance of Nutritional Therapy during CKD

The scientific community has shown a growing focus on the importance of nutritional therapy during CKD. In fact, nutritional therapy (in particular, VLPD) has demonstrated several beneficial effects in CKD patients, slowing the progression of CKD. Nutritional therapy has proven useful both for reducing protein intake and, consequently, the formation of urea and the urea symptoms associated with it, as well as providing the possibility of delaying the progression of CKD. Nutritional therapy is also useful in reducing the quantity and use of drugs in CKD patients, such as antihypertensive drugs, phosphate binders (to prevent secondary hyperparathyroidism and vascular calcification), erythropoietin (to treat anemia) and diuretics (to reduce edema) [183]. The objectives of nutritional therapy include the maintenance of an optimal nutritional status; the prevention and/or correction of signs, symptoms and complications of CKD; and, possibly, delaying the need for dialysis. Such diets include modulation of protein intake, adequate caloric intake, control of sodium and potassium intake and reduction of phosphorus intake [123]. Recent evidence has shown a connection between the kidneys and the intestines. In fact, increased urea levels in patients with CKD and favorable alteration of the gut microbiome can lead to an increase in permeability and alterations of the intestinal epithelial barrier [92]. For this reason, it is necessary to preserve the quality of the gut microbiome through the diet; namely by introducing the right amount of protein and fiber [184]. It has also been observed that a VLPD diet reduced PCS levels by about 30–35% in CKD patients after only one week of dietetic treatment [94]. Furthermore, evidence was presented that the uremic toxin reduction was due to a urea level decrease obtained by use of a nutritional therapy-derived plant. Similarly, supplementation of short-chain fatty acids (e.g., acetic acid, butyric acid, propionic acid and saturated fats) has shown a beneficial effect on inflammatory parameters and on gut-derived uremic toxins in HD patients [125].

## 6. Conclusions

The progression and severity of CKD are strongly associated with exacerbation of the inflammatory state and oxidative stress. These are known risk factors for the onset of serious systemic complications, cardiovascular disease, anemia and mineral disorders. Several studies have reported higher levels of pro-inflammatory enzymes, cytokines and oxidative stress markers, together with reduced antioxidative systems. Several biological mechanisms contribute to the onset and exacerbation of inflammation and oxidative stress, including mitochondrial activity, xanthine oxidase and NADPH oxidase. For these reasons, there has been a recent increase in scientific attention to natural substances with anti-inflammatory and antioxidant activities, which have demonstrated positive results in the treatment of CKD, both in vitro and *in vivo* (Table 3). Many of these compounds have been shown to reduce the inflammatory state by modulation of the NF-κB-dependent pathway, reducing the level of cytokines and pro-inflammatory enzymes. Other compounds have both direct ROS scavenging properties, due to their molecular structure or indirect antioxidative effects mediated by the upregulation of antioxidant enzymes (e.g., vitamin E, vitamin C, curcumin, resveratrol, green tea and other metabolites, flavonoids and polyphenols derived from plant species). However, the important limiting factors in most of these studies were low sample size, biodisponibility and short-term follow-up. As a consequence, none of these molecules have yet been introduced into clinical practice. Therefore, future prospective and comparative studies analyzing the co-administration of different anti-inflammatories and antioxidants with long-term follow-up are necessary.

## Figures and Tables

**Table 1:** Anti-inflammatory and antioxidant activities of the minerals (selenium and phosphate) and vitamins detailed in this study, with evaluation of the pathways involved. Decreased (↓) or increased (↑).

	*Anti-Inflammatory Activity*	*Antioxidant Activity*	*Experimental Model and References*
*↑Magnesium*	↓CRP		Human study; [84]
*↑Selenium*		↑GSH-Px ↓ROS	Human study; [87]
*↓* *Phosphate*	↓FGF23 ↓PTH		Human study; [90,92,123,124]
*↑* *Vitamin C*	↑hydroxylase/monooxygenase co-factor	↓MDA ↓tissue lipid oxidation ↑tissue GSH ↑Co-antioxidant vit.E ↓ROS	Human study; [98] Animal model; [99]
*↑* *Vitamin E*	↓8-OHdG	↓lipid peroxidation ↓NADPH activity ↓inflammatory mediators	Cellular and animal model; [101] Animal model; [102] Human study; [108]
*↑* *Vitamin D*	↓NF-kB signling pathway ↓RAAS ↓TGF-β/Smad ↓Wnt/β-catenin		Animal model; [114] Cellular and animal model; [115] Animal model; [116] Cellular and animal model; [117]
*↑* *Vitamin A*	↓TLR4/NF-kB signling pathway		Animal model; [118]
*↑* *Vitamin B1*		↓plasma lactate levels	Human study; [119]

8-OHdG, 8-hydroxy-2′ -deoxyguanosine; FGF 23, Fibroblast growth factor 23; GSH-Px, Glutathione peroxidase; MDA, Malondialdehyde; NADPH, Nicotinamide adenine dinucleotide phosphate hydrogen; NF-κB, nuclear factor kappa-light-chain-enhancer of activated B cells; PTH, Parathyroid Hormone; RAAS, renin–angiotensin–aldosterone system; ROS, Reactive oxygen species; TGF-β/Smad, Transforming growth factor-β/Smad; TLR4, Toll-like receptor 4; Vit. E, Vitamin E.

**Table 2:** The anti-inflammatory and antioxidant activities of the described plant-derived metabolites, with evaluation of the involved pathways which can result decreased (↓) or increased (↑).

	*Anti-Inflammatory Activity*	*Antioxidant Activity*	*Experimental Model and References*
*Quercetin*	↓FGF23 ↓PTH	↓LDH ↓SOD	Animal model; [130]
*Curcumin*	↓VEGF ↓TGF-β ↓CTGF ↓fibronectin and collagen IV ↓iNOS and ↓COX-2 ↓TNF-α ↓MCP-1 ↓JNK/NF-κB ↓NLRP3	↓NO and ↓ONOO^−^ ↓O_2_^−^ and ↓H_2_O_2_ ↑SOD ↑CAT ↑GSR ↑HO-1 ↑GST ↑NQO1 ↑GCL	Animal model; [131,132] Cellular model; [133,134,135,136,137] Animal model; [138,139,140,141]
*Resveratrol*	↓NF-κB ↓pro-inflammatory cytokines and enzymes	↑Nrf2 ↑antioxidant enzymes	Human study; [143,145] Animal model; [146,147,148]
*Cordycepin*	↓NF-κB ↓TNF-α ↓IL-6 ↓IL-1β ↓TGF-β1/Smad		Animal model; [153] Cellular model; [154] Human study and cellular model; [155]
*Flavonoids of C. tinctoria*	↓NF-κB signling pathway ↓COX-2 ↓MCP-1 and ↓collagen IV ↓AMPK ↓TGF-β/Smad		Animal model; [158,160] Cellular model; [161]
*Flavonoids and polyphenols of P. niruri*		↑SOD ↑CAT ↑GPx	Animal model; [164]
*Allicin*		↓AT1R ↓Keap1 ↓eNOS ↑Nrf2 ↑SOD ↑CAT ↑GPx	Animal model; [167,168]
*Ursolic acid*	↓IL-6 ↓NF-κB ↓p-STAT3 ↓C/EBP-δ		Human study and animal model; [170] Animal model; [172] Cellular and animal model; [173]
*Epigallocatechin-3-gallate*	↓NF-κB ↓NLRP3 ↓caspase-1 ↓IL-1β and IL-18 ↓p-Akt ↓p-JNK ↓p-ERK1/2 ↓p-P38 ↑PPARγ and ↑SIRT1 ↓MCP-1 ↓TGF-β	↑Nrf2 ↑GPx ↑HO-1 ↓AGE and lipid peroxidation ↓ROS	Cellular model; [176] Animal model; [177,178,179,180,181,182]

AGE, advanced glycation end-product; AMPK, 5′ AMP-activated protein kinase; AT1R, angiotensin II receptor type 1; C/EBP-δ, CCAAT-enhancer-binding proteins-δ; CAT, catalase; COX-2, cyclooxygenase-2; CTGF, connective tissue growth factor; eNOS, endothelial nitric oxide synthase; FGF 23, fibroblast growth factor 23; GCL, glutamate-cysteine ligase; GPx, glutathione peroxidase; GSR, glutathione-disulfide reductase; GST, glutathione S-transferase; H_2_O_2_, hydrogen peroxide; HO-1, heme oxygenase-1; IL-18, interleukin 18; IL-1β, interleukin 1β; IL-6, interleukin 6; iNOS, inducible nitric oxide synthase; JNK, c-Jun N-terminal kinases; KEAP1, Kelch-like ECH-associated protein 1; LHD, lactate dehydrogenase; MCP-1, monocyte chemoattractant protein-1; NF-κB, nuclear factor kappa-light-chain-enhancer of activated B cells; NLRP3, NOD-, LRR- and pyrin domain-containing protein 3; NO, nitric oxide; NQO1, NAD(P)H dehydrogenase [quinone] 1; Nrf2, nuclear factor erythroid 2-related factor 2; O_2_^−^, superoxide; ONOO^−^, peroxynitrite; p-Akb, phospho protein kinase B; p-ERK, phospho extracellular signal-related kinase; p-P38, phospho mitogen-activated protein kinases; PPARγ, peroxisome proliferator-activated receptor γ; p-STAT 3, phospho signal transducer and activator of transcription 3; PTH, parathyroid hormone; ROS, reactive oxygen species; SIRT-1, sirtuin 1; SOD, superoxide dismutase; TGF-β, transforming growth factor-β; TNF-α, tumor necrosis factor α; VEFG, vascular-endothelial growth factor.

**Table 3:** Potential contributions of the mentioned compounds in chronic kidney disease progression or complication management (summary table); “✓” indicates which of these aspects is modulated by the mentioned compounds.

	*Reduction of Progression of CKD*	*CKD-Complications Management*	*References*
*Magnesium*	✓	✓	[82,83,84,85]
*Selenium*		✓	[87]
*Phosphate*		✓	[89]
*Vitamin C*		✓	[96,97,98]
*Vitamin E*		✓	[102,103,104,105]
*Vitamin D*	✓	✓	[111,112,113,114,115,116,117]
*Vitamin A*	✓		[118]
*Vitamin B1*	✓	✓	[122]
*Quercetin*	✓		[129,130]
*Curcumin*	✓	✓	[131,132,133,134,135,136,137,138,139,140,141,142]
*Resveratrol*	✓	✓	[146,147,148,149]
*Cordycepin*	✓		[153,154,155]
*C. tinctoria*	✓		[160,161]
*P. niruri*	✓		[164]
*Allicin*	✓	✓	[166,167,168]
*Ursolic acid*	✓		[170,171,172,173,174,175]
*Epigallocatechin-3-gallate*	✓	✓	[177,178,179,180,181,182]

## Abbreviations

CKD	Chronic kidney disease
ESRD	End-stage renal disease
GFR	Glomerular filtration rate
Epo	Erythropoietin
CRP	C-reactive protein
IL-6	Interleukin-6
IL-1	Interleukin-1
TNF- α	Tumor necrosis factor-α
HD	Hemodialysis
MCP-1	Monocyte chemoattractant protein-1
PTH	Parathyroid hormone
RANKL	Receptor activator of NF-κB ligand
COX-2	Cyclooxygenase-2
iNOS	Inducible nitric oxide synthase
NF-κB	Nuclear factor kappa-light-chain-enhancer of activated B cells
IS	Indoxyl sulfate
PC	p-cresol
PCS	p-cresol sulfate
NO	Nitrogen monoxide
PD	Peritoneal dialysis
ROS	Reactive oxygen species
NOX	NADPH oxidases
8-OH-dG	8-hydroxy-2′-deoxyguanosine
RNS	Reactive nitrogen species
AGEs	Advanced glycation end products
RAGE	Receptor system for AGEs
Nrf2	Nuclear factor erythroid 2-related factor 2
SOD	Superoxide dismutase
CAT	Catalase
NQO1	NAD(P)H dehydrogenase [quinone] 1
MDA	Malondialdehyde
GSH-Px	Glutathione peroxidases
ATRA	All-trans retinoic acid
FGF23	Fibroblast growth factor 23
LDH	Lactate dehydrogenase
VEGF	Vascular endothelial growth factor
HEA	N6-(2-hydroxyethyl)-adenosine
UUO	Unilateral ureteral obstruction
EGCG	Epigallocatechin-3-gallate
VLPD	Very low protein diet
